# High-Energy Mode-Locked Pulse Er-Doped Fiber Laser-Based GeTe as Saturable Absorber

**DOI:** 10.3390/nano13162331

**Published:** 2023-08-14

**Authors:** Shouqian Tang, Qiuyan Sheng, Faming Ye, Qi Li, Siyuan Xiong, Caixun Bai, Cheng Lu, Huanian Zhang, Guomei Wang, Wenfei Zhang

**Affiliations:** 1 School of Physics and Optoelectronic Engineering, Shandong University of Technology, Zibo 255000, China; tangshouqian0728@163.com (S.T.); shengqiuyan0728@163.com (Q.S.); yfm2361354276@163.com (F.Y.); lq14494@163.com (Q.L.); 13875068547@163.com (S.X.); baicaixun@sdut.edu.cnn (C.B.); luchengcg@163.com (C.L.); huanian_zhang@163.com (H.Z.); 2 Shandong Ruixing Single Mode Laser Technology Co., Ltd., Zibo 255049, China

**Keywords:** high energy, high conversion efficiency, saturable absorber, mode-locked, GeTe

## Abstract

High-energy Er-doped fiber laser with high conversion efficiency is reported, which is mode-locked by a germanium telluride (GeTe)-based saturable absorber (SA). By adjusting the direction of the polarization controller (PC), a high-energy pulse with a central wavelength of 1533.1 nm and a fundamental repetition frequency of 1.58 MHz is achieved. Under the pump power of 450.1 mW, the maximum average output power is 50.48 mW, and the single-pulse energy is 32 nJ. It is worth noting that the optical-to-optical conversion efficiency has reached about 11.2%. The experimental results indicate that GeTe performs excellently as SAs for obtaining mode-locked fiber lasers and plays an extremely important role in high-energy fiber lasers.

## 1. Introduction

Passive mode-locked fiber lasers have the advantages of high conversion efficiency, low cost, compact structure, no collimation, and strong adaptability to the working environment, and they have a wide range of applications in many fields [1,2,3,4,5,6]. At present, the output of the mode-locked pulse is mainly achieved by adding a true SA or equivalent SA to the laser cavity [7,8,9,10,11]. In recent years, with the continuous development of laser basic theory and technology, high-energy fiber lasers have been widely used in basic research and core industrial production in various fields, such as micro-nano processing, ultrafast measurement, and biomedicine [12,13,14,15].

High-power pulsed fiber lasers have attracted attention because of their prospective applications in the fields of optical communications, biomedicine, industrial manufacturing, defense military, and nonlinear optics [16]. In general, because of the soliton area theorem, the traditional soliton pulse energy is limited to 0.1 nJ, which cannot be widely used in the direction of high energy demand. In order to solve this energy limitation, researchers have reported soliton rain, dissipative soliton resonance (DSR), and high-energy soliton pulses [17]. Among them, the high-energy soliton pulse has the characteristics of high peak power and energy and is favored in many research fields [18]. In 2019, Han et al. recorded a bidirectional pumped high-power laser based on In_2_Se_3_ as SA obtained single-pulse energy is 5.8 nJ at the pump power of 1324 mW [19]. In 2019, Fu et al. carried out research on high-energy fiber laser and obtained soliton mode-locked operation with the maximum single-pulse energy of 20.4 nJ [20]. In 2021, Zhu et al. realized a mode-locked fiber laser based on Cr_2_Si_2_Te_6_ as SA and obtained maximum average output power of 85.54 mW at the pump power of 1659 mW and single-pulse energy of 53.03 nJ [21]. A variety of SAs have been developed to fabricate high-energy pulsed fiber lasers. In the past few decades, the semiconductor saturable absorption mirror (SESAM) has been widely used in mode-locked fiber lasers as the most mature commercial saturable absorption device. However, SESAM has the drawbacks of narrow absorption bandwidth, a complicated preparation process, and an expensive price, which also limits its further application and development [22]. In recent years, with the emergence of graphene, various new two-dimensional (2D) materials have emerged. They are widely used in the research of passively mode-locked fiber lasers due to their excellent saturable absorption characteristics [23,24,25,26,27,28,29,30,31,32,33,34,35]. GeTe belongs to Ⅳ-Ⅵ semiconductor. Because of its phase change crystallization and high thermoelectricity of bilayer, it has attracted extensive attention from researchers. In 2021, Wang et al. studied the GeTe-based fiber laser and obtained the DSR. The maximum average single-pulse energy is 2.44 nJ [36]. The next year, it was reported that a conventional soliton mode-locked Er-doped fiber laser (EDFL) with a pulse width of 972 fs was realized based on GeTe SA [37]. Therefore, GeTe can be used as SAs to explore its performance in lasers. However, there is no report on the application of this material in high-energy pulse lasers.

As far as we know, the relaxation time is the second most important parameter in any SA, and this parameter strongly affects pulse parameters. In 2019, Guo et al. the n- and p-type thermoelectricity properties of GeTe by first-principles study [38]. The self-consistent single parabolic band (SPB) and deformation potential approximation (DPA) are used to evaluate the relaxation time. The relaxation time varies with temperature from 1.2 fs to 5.2 fs. In 2013, Levin et al. reported the electronic and thermal transport in GeTe: A versatile base for thermoelectric materials [39]. Spin-lattice relaxation time, T_1_, was determined by fitting the dependence of normalized integral and time delay after saturation by a train of 90° pulses. This work achieved a relaxation time of 5.3 ms. In 2021, Cui et al. focus on r-GeTe, a low-temperature phase of GeTe, and investigate the pressure effects on the electronic structure, electrical transport properties, and anharmonic lattice dynamics based on density functional theory (DFT), the Boltzmann transport equations (BTEs) and perturbation theory [40]. Based on electron–phonon interaction and constant relaxation time approximation, an electron relaxation time of 10 fs was obtained.

In our work, we reveal a high-power pulsed fiber laser based on GeTe-PVA film as SA. By optimizing the output port parameters. The maximum energy can be obtained when the output ratio is 80%. In this state, we obtained a stable large energy mode-locked pulse with a maximum average output power of 50.48 mW and a fundamental repetition frequency of 1.58 MHz. Under the pump power of 450.1 mW, the maximum average output power is 50.48 mW, and the corresponding optical-to-optical conversion efficiency is 11.2%. Our work has more forcefully proved the excellent performance of GeTe as SAs, and revealed its unique achievements in the field of high-power pulse.

## 2. Preparation and Characterization of GeTe SA

### 2.1. Crystal Model of GeTe

**Figure 1:**
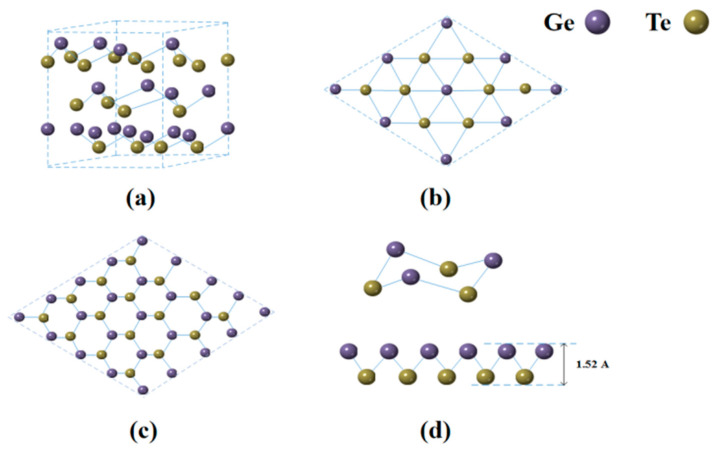
Crystal model of GeTe. (**a**) 3D diagram; (**b**) Top view; (**c**) Side view; (**d**) Single crystal structure diagram.

GeTe is a binary main group sulfur compound with three main crystalline forms: α crystalline phase (rhombohedral hexahedron) at room temperature, γ crystalline phase (rhombohedral crystal system), and β crystalline phase (cubic crystal phase) at high temperature. Among them, GeTe at room temperature is the most stable and common crystalline phase with space group R3m and lattice constants: a = 8.343 Å, b = 8.343 Å, c = 10.668 Å; α = β = 90°, γ = 120°. Figure 1 represents the supercell structure of α-GeTe crystals and the configuration of α-GeTe crystals at different angles. The figure shows that the optimized bulk α-GeTe crystal has a layer spacing of 3.58 Å, a bond length of 2.86 Å, and a bond angle of 95.4°. However, lattice distortion occurs when the number of layers of the material is reduced to a single layer, causing some changes in the bond length and angle to 2.77 Å and 91.17°, respectively [41,42,43].

### 2.2. Characterization of GeTe

**Figure 2:**
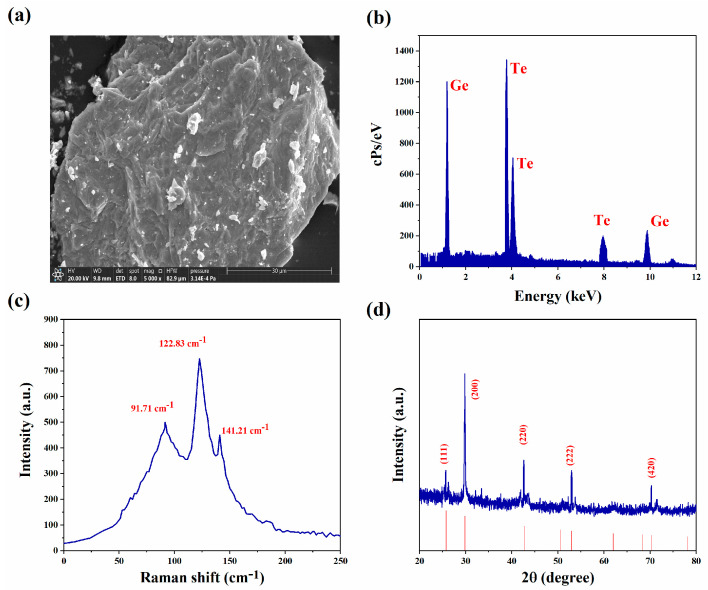
Characterizations of GeTe. (**a**) SEM image; (**b**) EDS spectrum; (**c**) Raman spectrum; (**d**) XRD.

In order to test the GeTe nanosheets prepared in this experiment, we used a variety of techniques to characterize and analyze their different characteristics. Figure 2a shows the surface morphology of GeTe. At the resolution of 30 μm, the obvious layered structure is shown in a scanning electron microscopy (SEM) image. The energy dispersive spectroscopy (EDS) is depicted in Figure 2b. It can be seen that Ge and Te existed in the measured sample. Therefore, it confirms the high purity of the GeTe nanosheets [44,45]. In the Raman spectrum, as provided in Figure 2c, there are three obvious peaks located at 91.71, 122.83, and 141.21 cm^−1^, respectively. The crystal structure of the GeTe nanosheets is also studied by X-ray diffraction (XRD), as is shown in Figure 2d. The diffraction peaks at (111), (200), (220), (222), and (420) planes in GeTe, respectively.

**Figure 3:**
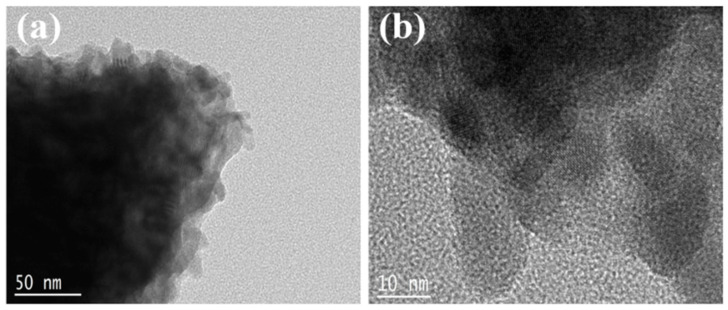
TEM image of GeTe nanosheets.(**a**) TEM image and (**b**) HR-TEM images of GeTe nanosheets.

Figure 3 indicates the transmission electron microscopy (TEM) image of GeTe nanosheets. It can be seen from Figure 3a that the obvious layered structure of GeTe is recorded under a resolution of 50 nm. Figure 3b shows the image of GeTe nanosheets at 10 nm. It can be seen that the nanosheets are very transparent.

### 2.3. Preparation of GeTe SA

**Figure 4:**
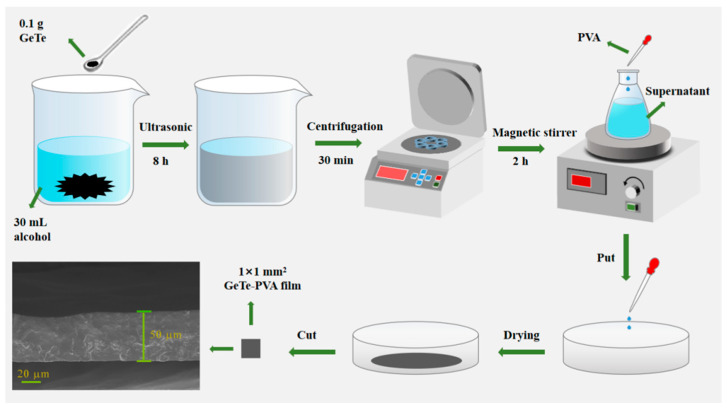
Preparation processes of few-layer GeTe nanosheets and GeTe SA.

The GeTe nanosheets are fabricated using the liquid-phase exfoliation (LPE) method, which is a direct method of exfoliating layered structural materials. As shown in Figure 4, firstly, 0.1 g GeTe nanosheets are mixed with 30 mL alcohol and subjected to ultrasonic treatment for 8 h to prepare a GeTe dispersion solution. Then, the GeTe-alcohol is centrifuged for 30 min, which is an essential step to remove the precipitation. Next, 5 wt.% polyvinyl alcohol (PVA) is added to the GeTe supernatant solution using a magnetic stirrer. After that, the blended solution is placed on a smooth glass substrate and evaporated at a super clean room for 72 h to obtain GeTe-PVA thin film. Subsequently, divide GeTe film into 1 × 1 mm^2^ small pieces and insert it into optical fiber as SA. Finally, GeTe SA is prepared successfully. The SEM image of the prepared GeTe-PVA thin film is shown in the inset. It can be seen that the thickness of the film is 50 μm.

**Figure 5:**
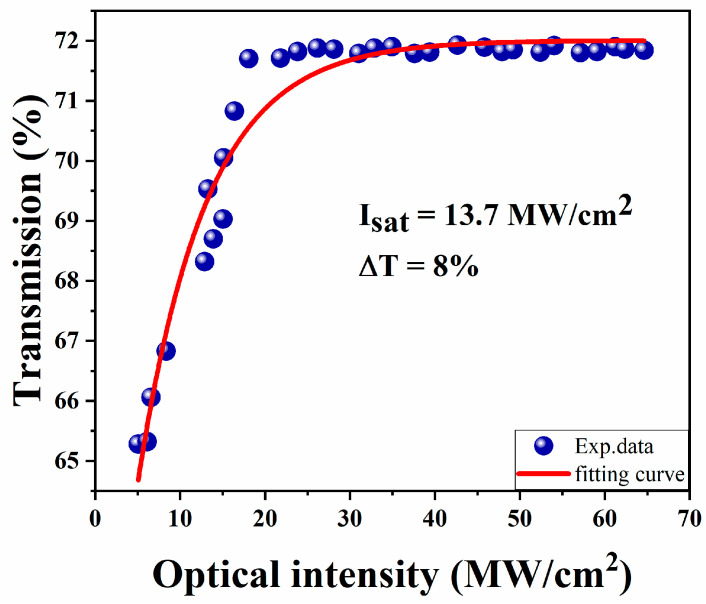
Nonlinear absorption property of GeTe SA.

The twin-detection technique is employed to study the nonlinear optical characteristics of GeTe-PVA films. The repetition frequency, center wavelength, and pulse width of the laser source are 23.74 MHz, 1562.28 nm, and 464.47 fs, respectively. The non-linear fitting curve is shown in Figure 5. The experiment data are fitted by

(1) $$T\left(I\right)=1-T_{ns}-\Delta T\cdot exp(-I/I_{sat})\;$$

where *I* is input pulse energy, *T*(*I*) is transmission rate, $I_{sat}$ is saturation intensity, Δ*T* is modulation depth, $T_{ns}$ is nonsaturable loss. Through fitting, the saturation intensity and modulation depth can be estimated as 13.7 MW/cm^2^ and 8%, respectively.

## 3. Experimental Setup

**Figure 6:**
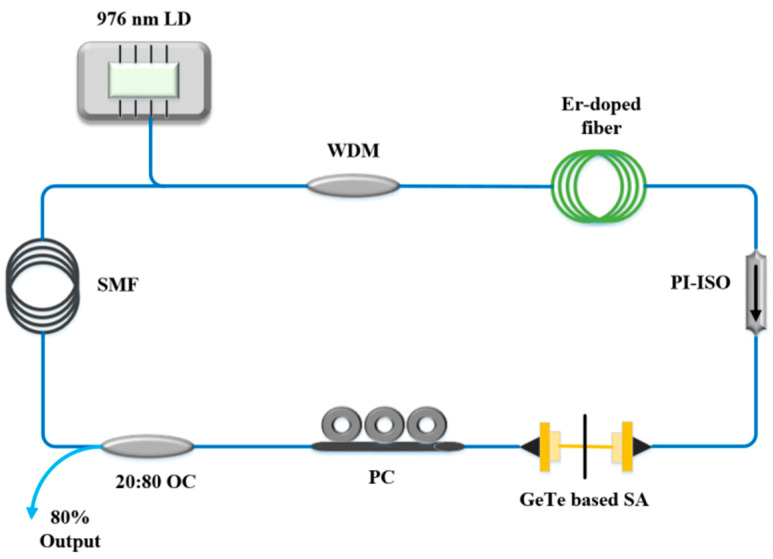
Experimental setup of the passively mode-locked fiber laser with GeTe SA.

The experimental device of the mode-locked EDFL is provided in Figure 6. The pump adopts a 976 nm laser diode (LD) and is connected to the cavity through 980/1550 nm Wavelength division multiplexing (WDM). The gain medium is EDF, whose length is 8 m, and its dispersion parameter is −18 ps/nm/km. A 110 m ordinary single-mode fiber (SMF) is used to control the dispersion in the cavity, and the whole ring cavity is about 128.7 m. The GeTe-PVA film as SA was inserted between the polarization-independent isolator (PI-ISO) and PC to realize the mode-locked operation. The insertion loss of the GeTe saturable absorber is approximately 1.8 dB. In addition, a 20:80 OC is chosen for this fiber laser, 80% of the signal is input to the measurement equipment, and the remained signal continues to operate to ensure the continuity of the laser.

## 4. Experimental Results and Discussions

The optical pulses oscillate in the ring cavity laser continuously. They are periodically affected by the gain, the loss, the dispersion, and the nonlinear effects, so their pulse width, energy, and other parameters change constantly during the oscillation process in the laser [46]. Therefore, the settings of the fiber laser can be optimized to improve its output performance of the laser. Table 1 summarizes the output performance of the lasers at different coupling ratios. It can be seen that the different output coupling proportion of the laser has a greater impact on its output performance. The larger the output proportion of the coupler, the greater the loss of optical pulse when passing through the coupler, which leads to a higher laser mode-locked threshold. At the same time, by comparing the output performance of five different ratios, it is found that the laser has the best performance when the output ratio is 80%. In this case, the laser can obtain stable mode-locked output from the pump power of 100.4 to 450.1 mW. When the maximum pump power is 450.1 mw, the single-pulse energy is 32 nJ. At 80% output coupling ratio, the conversion efficiency reaches 11.2% when the pump power is 450.1 mW.

**Figure 7:**
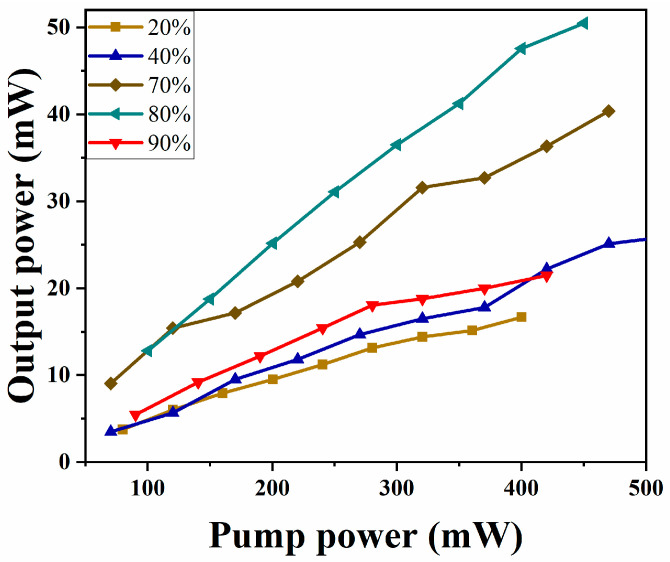
The relationship between the average output power and the pump power of the pulsed laser with different output ratios.

In order to further characterize the output performance of the pulsed laser, we test the output power of the laser under different output coupling ratios, as shown in Figure 7. It can be seen that the different coupling ratio of laser output not only affects the laser threshold but also affects the conversion efficiency of pump light and signal light. When the pump power increase, the output energy continues to increase. At the OC ratio of 20%, 40%, 70%, 80%, and 90%, the corresponding maximum average output power is 16.6 mW, 26 mW, 40.38 mW, 50.48 mW, and 21.46 mW, respectively. It can be seen that at an 80% output coupling ratio, the maximum single-pulse energy is 32 nJ. Therefore, at an 80% output coupling ratio, its single pulse energy and conversion efficiency are the highest.

**Figure 8:**
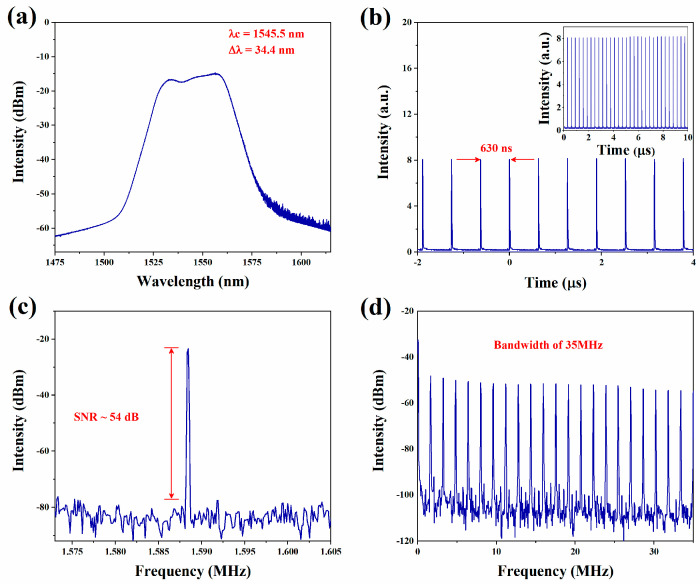
Output characteristics of the fiber laser at the pump power of 450 mW. (**a**) The output optical spectrum; (**b**) The pulse train of the mode-locked operation; (**c**) RF spectrum; (**d**) RF spectrum within a bandwidth of 35 MHz.

In this work, we recorded the features of the laser not inserting the GeTe-PVA films. By tuning the pump power and PC, no pulse was generated in the cavity, indicating that the laser had not appeared in self-mode-locked operation. Then, the GeTe-PVA film is interposed into the cavity. By measurement, the PDL is approximately 0.22 dB. At an 80% output coupling ratio, when increasing the pump power to 100.4 mW and adjusting the PC, a mode-locked operation can be acquired with an average output power of 12.8 mW. And the single-pulse energy is 8.1 nJ. As recorded in Figure 8a, the central wavelength is 1545.5 nm, and the 3-dB bandwidth is 34.4 nm. Figure 8b demonstrates the pulse train of mode-locked operation. The pulse-to-pulse distance is 630 ns, corresponding to cavity length. In addition, the inset shows more mode-locked pulse trains, which proves the stability of the mode-locked operation. To further test the stability of the mode-locked operation, we also observed its RF spectrum, as illustrated in Figure 8c. A signal-to-noise ratio (SNR) greater than 50 dB indicates that the operation is in a relatively stable state. Figure 8d provides the RF spectrum within a 35 MHz bandwidth, which further explains the high constancy of the laser mode-locked state.

**Figure 9:**
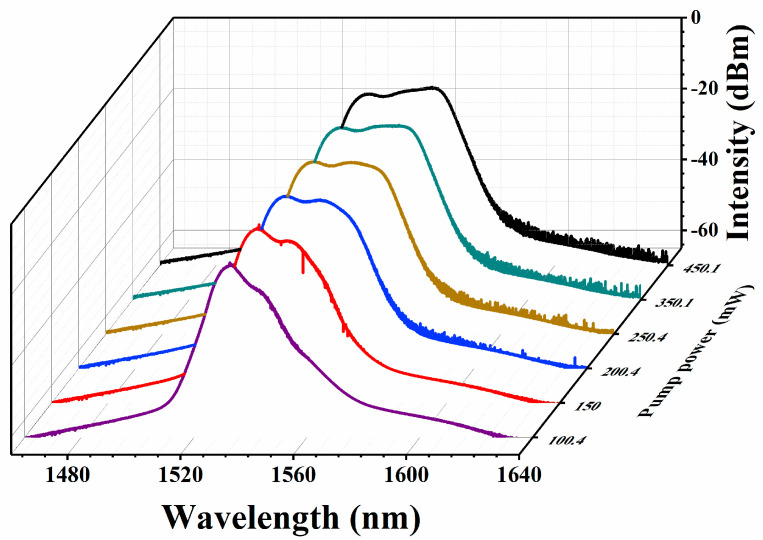
Output optical spectrum at different pump power.

Additionally, we also tested the stability of the mode-locked laser and observed the spectra at different pump powers. The output optical spectra are observed in Figure 9. It can be seen that the width of the optical spectra broadens with the increase of the pump power. Meanwhile, as the pump power increases from 100.4 to 450.1 mW, the 3-dB bandwidth of the spectrum increases from 9 to 34 nm. By comparing with other relevant references, it was found that the high-energy mode-locked pulse may be a noise-like pulse [17,47,48].

**Figure 10:**
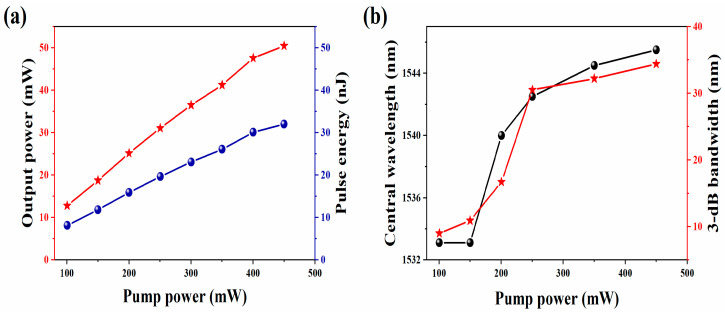
(**a**) The relationship between the average output power, single-pulse energy, and the pump power; (**b**) The central wavelength and 3-dB bandwidth vary with the pump power.

As shown in Figure 10a, with the increase of the pump power from 100.4 to 450.1 mW, the output power and pulse energy increase. When the pump power continues to increase from 450.1 mW, the pulse splitting phenomenon occurs. Figure 10b indicates the relationship between the central wavelength, 3-dB bandwidth, and the pump power.

**Figure 11:**
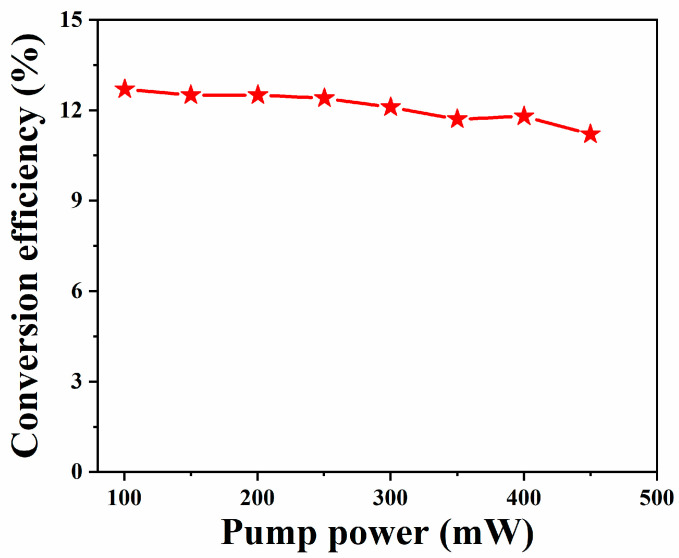
Relationship between pump power and conversion efficiency.

Figure 11 shows the relationship between the pump power and the conversion efficiency. It can be observed that with the increase of the pump power, the conversion efficiency decreases slightly from 12.7% to 11.2%. In this case, as the pump power gradually increases, the intra-cavity loss also increases, which may lead to a decrease in the optical-to-optical conversion efficiency.

Table 2 summarizes the performance of high-power mode-locked lasers using different 2D materials as SA. The pump power, center wavelength, SNR, pulse duration, average output power single pulse energy, and conversion efficiency of each laser are compared. It is obvious that the single-pulse energy of 32 nJ obtained at the pump power of 450.1 mW is already extremely high. It can be calculated that the optical-to-optical conversion efficiency is 11.2%, which is far greater than the efficiency value of those fiber lasers based on other 2D materials. In addition, it can be seen that the SNR based on GeTe-SA is 54 dB, which is higher than materials such as In_2_Se_3_, InSe, MoS_2_, and Bi_2_Te_3_, and shows good stability performance. The pulse duration is wider than that of tellurite and Bi_2_Te_3_, but the obtained pulse energy for mode-locked operation is greater than these. In future work, we will conduct in-depth research on the manufacture of SA, which enables the materials to work stably at higher pump power and obtain higher conversion efficiency.

## 5. Conclusions

In this work, an EDFL based on GeTe as SA is studied, and high energy mode-locked pulse operation is obtained. The saturation intensity and modulation depth can be estimated as 13.7 MW/cm^2^ and 8%, respectively. By adjusting the direction of the PC, a large energy pulse with a central wavelength of 1533.1 nm and a fundamental repetition frequency of 1.58 MHz is acquired. Under the pump power of 450.1 mW, the maximum average output power is 50.48 mW, and the single-pulse energy is 32 nJ. In this case, the optical-to-optical conversion efficiency is about 11.2%. Through consulting the papers, it can be found that it is very rare to reach such large single-pulse energy at a similar pump power level. The experimental results indicate that GeTe has unique achievements in acting as SAs for obtaining high-energy fiber laser operations.

## Figures and Tables

**Table 1:** The output performance of the laser at different coupling ratios.

Optical Pulse Output Ratio (%)	Pulse Splitting Threshold (mW)	Maximum Single-Pulse Energy (nJ)	Conversion Efficiency (%)
20	400.2	10.69	4.1
40	520.4	16.7	4.9
70	470	26.05	8.6
80	450.1	32	11.2
90	420.3	13.78	5.1

**Table 2:** Comparison of high energy mode-locked lasers based on different SAs.

SAs	Pump Power (mW)	Wavelength (nm)	SNR(dB)	Pulse Duration (ns)	Output Power (mW)	Pulse Energy (nJ)	Conversion Efficiency (%)	Reference
tellurene	1120	1563.97	55	0.059	106.6	8.76	9.25	[17]
In_2_Se_3_	1324	1559.4	42	14.4	122.4	5.8	9.24	[19]
InSe	560	-	45	389.2	11.96	20.4	2.13	[20]
MoS_2_	1725	1564.58	50	10.84	122.77	130.49	7.1	[49]
Bi_2_Te_3_	659	1558.459	50	3.22	40.7	23.9	6.17	[50]
ZrS_2_	1250	1531	/	181	14.19	23.65	1.14	[51]
GeTe	450.1	1545.5	54	8.3	50.48	32	11.22	Ours

## Data Availability

Data underlying the results presented in this paper are not publicly available at this time but may be obtained from the authors upon reasonable request.

## References

[1] Rashi F.A.A., Azzuhri S.R., Salim M.A.M., Shaharuddin R.A., Ismail M.A., Ismail M.F., Razak M.Z.A., Ahmad H. (2016). Using a black phosphorus saturable absorber to generate dual wavelengths in a Q-switched ytterbium-doped fiber laser. Laser Phys. Lett..

[2] Shang X.X., Guo L.G., Zhang H.N., Li D.W., Yue Q.Y. (2020). Titanium disulfide based saturable absorber for generating passively mode-locked and Q-switched ultra-fast fiber lasers. Nanomaterials.

[3] Xu N.N., Sun S., Shang X.X., Zhang H.N., Li D.W. (2022). Harmonic and fundamental-frequency mode-locked operations in an Er-doped fiber laser using a Cr_2_Si_2_Te_6_-based saturable absorber. Opt. Mater. Express.

[4] Shang X.X., Xu N.N., Zhang H.N., Li D.W. (2022). Nonlinear photoresponse of high damage threshold titanium disulfide nanocrystals for Q-switched pulse generation. Opt. Laser Technol..

[5] Liu S.C., Wang Y.G., Lv R.D., Wang J., Wang H.Z., Wang Y., Duan L.N. (2020). 2D molybdenum carbide (Mo_2_C)/fluorine mica (FM) saturable absorber for passively mode-locked erbium-doped all-fiber laser. Nanophotonics.

[6] Chen B., Zhang X.Y., Wu K., Wang H., Wang J. (2015). Q-switched fiber laser based on transition metal dichalcogenides MoS_2_, MoSe_2_, WS_2_, and WSe_2_. Opt. Express.

[7] Du J., Wang Q.K., Jiang G.B., Xu C.W., Zhao C.J., Xiang Y.J., Chen Y., Wen S.C., Han Z. (2014). Ytterbium-doped fiber laser passively mode locked by few-layer Molybdenum disulfide (MoS_2_) saturable absorber functioned with evanescent field interaction. Sci. Rep..

[8] Luo Z.C., Liu M., Liu H., Zheng X.W., Luo A.P., Zhao C.J., Zhang H., Wen S.C., Xu W.C. (2013). 2 GHz passively harmonic mode-locked fiber laser by a microfiber-based topological insulator saturable absorber. Opt. Lett..

[9] Zhao C.J., Zou Y.H., Chen Y., Wang Z.T., Tang D.Y. (2013). Wavelength-tunable picosecond soliton fiber laser with Topological Insulator: Bi_2_Se_3_ as a mode locker. Opt. Express.

[10] Guo B., Yao Y., Yang Y.F., Yuan Y.J., Jin L., Yan B., Zhang J.Y. (2015). Dual-wavelength rectangular pulse erbium-doped fiber laser based on topological insulator saturable absorber. Photonics Res..

[11] Gusev G.M., Kvon Z.D., Shegai O.A., Mikhailov N.N., Dvoretsky S.A., Portal J.C. (2011). Transport in disordered two-dimensional topological insulator. Phys. Rev. B.

[12] Hu N.N., Zhang H.N., Man B.Y. (2018). Various large-energy soliton operations within an Er-doped fiber laser with bismuth selenide as a saturable absorber. Appl. Opt..

[13] Zhang H., Bao Q.L., Tang D.Y., Zhao L.M., Loh K.P. (2009). Large energy soliton erbium-doped fiber laser with a graphene-polymer composite mode locker. Appl. Phys. Lett..

[14] Li F., Zhao W., Wang Y.S., Li D.J., Song D.D., Li Q.L., Yang Y., Wen W.L. (2023). Large dispersion-managed broadband high-energy fiber femtosecond laser system with sub 300 fs pulses and high beam quality output. Opt. Laser Technol..

[15] Li X.H., Liu X.M., Hu X.H. (2010). Long-cavity passively mode-locked fiber ring laser with high-energy rectangular-shape pulses in anomalous dispersion regime. Opt. Lett..

[16] Li L., Pang L.H., Wang R.F., Zhang X.G., Hui Z.Q., Han D.D., Zhao F., Liu W.J. (2022). Ternary Transition Metal Dichalcogenides for High Power Vector Dissipative Soliton Ultrafast Fiber Laser. Laser Photonics Rev..

[17] Xu N.N., Ma P.F., Fu S.G., Shang X.X., Jiang S.Z., Wang S.Y., Li D.W., Zhang H.N. (2020). Tellurene-based saturable absorber to demonstrate large-energy dissipative soliton and noise-like pulse generations. Nanophotonics.

[18] Xu N.N., Ming N., Han X.L., Man B.Y., Zhang H.N. (2019). Large-energy passively Q-switched Er-doped fiber laser based on CVD-Bi_2_Se_3_ as saturable absorber. Opt. Mater. Express.

[19] Han X.L., Zhang H.N., Jiang S.Z., Zhang C., Li D.W., Guo Q.X., Gao J.J., Man B.Y. (2019). Improved Laser Damage Threshold of In_2_Se_3_ Saturable Absorber by PVD for High-Power Mode-Locked Er-Doped Fiber Laser. Nanomaterials.

[20] Fu S.G., Li J.J., Zhang S.S., Bai Z.D., Wu T.G., Man Z.S. (2019). Large-energy mode-locked Er-doped fiber laser based on indium selenide as a modulator. Opt. Mater. Express.

[21] Zhu M.X., Yang F.H., Sun S., Chen S., Wang Y.J., Sui Z.Q., Hong Z.F., Wang G.M., Zhang W.F., Zhang H.H. (2021). Large-energy mode-locked Er-doped fiber laser based Cr_2_Si_2_Te_6_ as a modulator. Infrared Phys. Technol..

[22] Okhotnikov O., Grudinin A., Pessa M. (2004). Ultra-fast fibre laser systems based on SESAM technology: New horizons and applications. New J. Phys..

[23] Sotor J., Sobon G., Macherzynski W., Paletko P., Abramski K.M. (2015). Black phosphorus saturable absorber for ultrashort pulse generation. Appl. Phy. Lett..

[24] Xu Y.J., Shi Z., Shi X.Y., Zhang K., Zhang H. (2019). Recent progress in black phosphorus and black-phosphorus-analogue materials: Properties, synthesis and applications. Nanoscale.

[25] Niu K.D., Chen Q.Y., Sun R.Y., Man B.Y., Zhang H.N. (2017). Passively Q-switched erbium-doped fiber laser based on SnS_2_ saturable absorber. Opt. Mater. Express.

[26] Orchin G.J., Fazio D.D., Bernardo A.D., Hamer M., Yoon D., Cadore A.R., Goykhman I., Watanabe K., Taniguchi T., Robinson J.W.A. (2019). Niobium diselenide superconducting photodetectors. Appl. Phys. Lett..

[27] Li Z.Q., Dong N.N., Zhang Y.X., Wang J., Yu H.H., Chen F. (2018). Mode-locked waveguide lasers modulated by rhenium diselenide as a new saturable absorber. APL Photonics.

[28] Lan D.F., Cheng T.L., Qu Y.H., Zhang X.N., Yan X., Suzuki T., Ohishi Y., Wang F. (2022). Tungsten Carbide Nanoparticles as Saturable Absorber for Q-Switched Erbium-Doped Fiber Laser. IEEE Photonics Technol. Lett..

[29] Wang X.D., Luo Z.C., Liu H.M., Luo A.P., Xu W.C. (2014). Microfiber-based gold nanorods as saturable absorber for femtosecond pulse generation in a fiber laser. Appl. Phys. Lett..

[30] Zhang M., Kelleher E., Torrisi F., Sun Z., Hasan T., Popa D., Wang F., Ferrari A.C., Popov S.V., Taylor J.R. (2012). Tm-doped fiber laser mode-locked by graphene-polymer composite. Opt. Express.

[31] Jiang Z.K., Chen H., Li J.R., Yin J.D., Wang J.Z., Yan P.G. (2017). 256 fs, 2 nJ soliton pulse generation from MoS_2_ mode-locked fiber laser. Appl. Phys. Express.

[32] Yang S., Yang Y.Y., Zhang L., Huang J.Y., Bai Y.R., Lin X.C. (2019). 25 nJ, 634 ps and 1 MHz all-fiber passively mode-locked fiber laser based on a GaAs saturable absorber. Optik.

[33] Luo Z.Q., Zhou M., Weng J., Huang G.M., Xu H.Y., Ye C.C., Cai Z.P. (2010). Graphene-based passively Q-switched dual-wavelength erbium-doped fiber laser. Opt. Lett..

[34] Chen Y., Zhao C.J., Chen S.Q., Du J., Tang P.H., Jiang G.B., Tang D.Y. (2014). Large energy, wavelength widely tunable, topological insulator Q-switched erbium-doped fiber laser. IEEE J. Quantum Electron..

[35] Baranava M.S., Hvazdouski D.C., Skachkova V.A., Stempitsky V.R., Danilyuk A.L. (2020). Magnetic interactions in Cr_2_Ge_2_Te_6_ and Cr_2_Si_2_Te_6_ monolayers: Ab initio study. Mater. Today Proc..

[36] Wang G.M., Zhang W.F., Han K.Z., Xing F., Zhang H.N., Fu S.G. (2021). Q-switched dissipative soliton resonance operation in GeTe based fiber laser. Infrared Phys. Technol..

[37] Wang G.M., Zhang W.F., Han K.Z., Lu C., Zhang H.N., Fu S.G. (2022). GeTe based modulator for the generation of soliton, soliton molecule and bright-dark soliton pair. Infrared Phys. Technol..

[38] Guo D.L., Li C.H., Qiu K.B., Yang Q.Q., Li K.J., Shao B., Chen D.M., Ma Y.L., Sun J.C., Cao X.L. (2019). The n- and p-type thermoelectricity property of GeTe by first-principles study. J. Alloys Compd..

[39] Levin E.M., Besser M.F., Hanus R. (2013). Electronic and thermal transport in GeTe: A versatile base for thermoelectric materials. J. Appl. Phys..

[40] Cui J., Li S.S., Xia C.X., Chen Y., He J.Q. (2021). Pressure effects on the electrical transport and anharmonic lattice dynamics of r-GeTe: A first-principles study. J. Mater..

[41] Dong J.F., Sun F.H., Tang C., Pei J., Zhuang H.L., Hu H.H., Zhang B.P., Pan Y., Li J.F. (2019). Medium-temperature thermoelectric GeTe: Vacancy suppression and band structure engineering leading to high performance. Energy Environ. Sci..

[42] Zhang P.P., Zhao F.L., Long P., Wang Y., Yue Y.C., Liu X.Y., Feng Y.Y., Li R.J., Hu W.P., Li Y. (2018). Sonication-assisted liquid-phase exfoliated α-GeTe: A two-dimensional material with high Fe_3_ sensitivity. Nanoscale.

[43] Liu W.D., Wang D.Z., Liu Q.F., Zhou W., Shao Z.P., Chen Z.G. (2020). High-Performance GeTe-Based Thermoelectrics: From Materials to Devices. Adv. Energy Mater..

[44] Cheng L., Yuan Y.F., Liu C.M., Cao X.R., Su J., Zhang X.T., Zhang H., Zhao H.B., Xu M., Li J. (2019). Linear and nonlinear optical properties modulation of Sb_2_Te_3_/GeTe bilayer film as a promising saturable absorber. Results Phys..

[45] Sheng Q.Y., Tang S.Q., Ye F.M., Wang Y.J., Chen S., Bai C.X., Lu C., Zhang H.N., Fu S.G., Wang G.M. (2022). Passively mode-locked fiber laser based on GeTe as a saturable absorber. Appl. Opt..

[46] Haus H.A. (2000). Mode-locking of lasers. IEEE J. Sel. Top. Quantum Electron..

[47] Zheng X.W., Luo Z.C., Liu H., Zhao N., Ning Q.Y., Liu M., Feng X.H., Xing X.B., Luo A.P., Xu W.C. (2014). High-energy noiselike rectangular pulse in a passively mode-locked figure-eight fiber laser. Appl. Phys. Express.

[48] Wang X.F., Xia Q., Gu B. (2018). A 1.9 μm noise-like mode-locked fiber laser based on compact figure-9 resonator. Opt. Commun..

[49] Ma P.F., Lin W., Zhang H.N., Xu S.H., Yang Z.M. (2019). High-Power Large-Energy Rectangular Mode-Locked Er-Doped Fiber Laser Based on High-Damage-Threshold MoS_2_ Saturable Absorber. IEEE Photon..

[50] Wei Q., Niu K.G., Han X.L., Zhang H.N., Zhang C., Yang C., Man B.Y. (2019). Large energy pulses generation in a mode-locked Er-doped fiber laser based on CVD-grown Bi_2_Te_3_ saturable absorber. Opt. Mater. Express.

[51] Sui Z.Q., Yang F.H., Han Y.A., Fan W.Y., Li S.M., Bai C.X., Lu C., Zhang W.F., Wang G.M., Fu S.G. (2023). Large energy mode-locked phenomenon based on ZrS_2_ in Er-doped fiber laser. Opt. Laser Technol..

